# Circadian Rhythmicity of Antioxidant Markers in Rats Exposed to 1.8 GHz Radiofrequency Fields

**DOI:** 10.3390/ijerph120202071

**Published:** 2015-02-12

**Authors:** Honglong Cao, Fenju Qin, Xueguan Liu, Jiajun Wang, Yi Cao, Jian Tong, Heming Zhao

**Affiliations:** 1 School of Electronic & Information Engineering, Soochow University, Suzhou 215006, China; E-Mails: caohonglong@suda.edu.cn (H.C.); txdzlxg@suda.edu.cn (X.L.); jjwang@suda.edu.cn (J.W.); 2 Department of Biological Science and Technology, Suzhou University of Science and Technology, Suzhou 215009, China; E-Mail: qinfenju@163.com; 3 School of Public Health, Medical College of Soochow University, Suzhou 215123, China; E-Mails: yicao@suda.edu.cn (Y.C.); tongjian@suda.edu.cn (J.T.)

**Keywords:** radio frequency, circadian rhythmicity, antioxidant markers, Mel, SOD, GSH-Px

## Abstract

Background: The potential health risks of exposure to Radiofrequency Fields (RF) emitted by mobile phones are currently of considerable public interest, such as the adverse effects on the circadian rhythmicities of biological systems. To determine whether circadian rhythms of the plasma antioxidants (Mel, GSH-Px and SOD) are affected by RF, we performed a study on male Sprague Dawley rats exposed to the 1.8 GHz RF. Methods: All animals were divided into seven groups. The animals in six groups were exposed to 1.8 GHz RF (201.7 μW/cm^2^ power density, 0.05653 W/kg specific absorption rate) at a specific period of the day (3, 7, 11, 15, 19 and 23 h GMT, respectively), for 2 h/day for 32 consecutive days. The rats in the seventh group were used as sham-exposed controls. At the end of last RF exposure, blood samples were collected from each rat every 4 h (total period of 24 h) and also at similar times from sham-exposed animals. The concentrations of three antioxidants (Mel, GSH-Px and SOD) were determined. The data in RF-exposed rats were compared with those in sham-exposed animals. Results: circadian rhythms in the synthesis of Mel and antioxidant enzymes, GSH-Px and SOD, were shifted in RF-exposed rats compared to sham-exposed animals: the Mel, GSH-Px and SOD levels were significantly decreased when RF exposure was given at 23 and 3 h GMT. Conclusion: The overall results indicate that there may be adverse effects of RF exposure on antioxidant function, in terms of both the daily antioxidative levels, as well as the circadian rhythmicity.

## 1. Introduction

The rapid development and deployment of wireless communication devices such as mobile phones have made a positive impact in modern society although the general public is concerned about their “potential” negative influence on human health due to reports suggesting a link between the radio frequency field (RF) emitted from mobile phones and increased genotoxicity, brain tumor development and neurodegenerative diseases [1,2,3,4,5,6,7]. Adverse effects reported in some studies were attributed when the specific absorption rate (SAR) from RF exposure exceeded 4 W/kg resulting in hyperthermia/increased temperature. Based on such observations, the International Commission on Non-Ionizing Radiation [8] has recommended certain guidelines to protect the occupationally exposed as well as the general public to avoid excess exposure to RF. However, in recent years, evidence is emerging on non-thermal biological effects of RF exposures at different frequencies and SARs/power densities [9,10,11,12].

There are, however, very few reports thus far taking into account circadian associated effects from RF radiation due to mobile phone exposure on different biological systems, especially on the oxidative damage. Reactive oxygen species (ROS) were reported to be directly involved in causing oxidative damage in cellular macromolecules such as lipids, proteins, and nucleic acids in tissues leading to oxidative stress which was suggested to play an important role in several human health conditions such as atherosclerosis, cardiovascular diseases, neurodegenerative disorders, cancer and the aging process [13]. Nonetheless, cells have developed protective mechanisms to scavenge ROS through the production of antioxidant enzymes such as glutathione peroxidase (GSH-Px), superoxide dismutase (SOD), catalase (CAT), *etc*. However, when ROS generation is outweighed, the endogenous antioxidative defense system is likely to be perturbed [14]. Recent reports have also suggested that melatonin (Mel) is a potent anti-oxidant that exerts many receptor-mediated and receptor-independent activities [15] and regulates the expression of several genes involved in the production of numerous antioxidant enzymes [16]. In mammals, Mel is synthesized by the pineal gland in the brain in a circadian rhythm and was reported to play an important role in physiological detoxification of ROS and thus, acts as an antioxidant [17,18,19].

Since people generally hold mobile phones near to head when they are making a phone call or answering the phone, their brains are being exposed more to RF than other parts of the body, many studies have reported that people using mobile phone present with changed levels of Mel. But thus far, there were controversial reports, in animals and humans, indicating a decrease, no significant change or an increase in Mel synthesis as well as antioxidant levels following exposure to RF [20,21,22,23,24,25]. Although preliminary results of these studies have shown that RF may change antioxidant levels, as well as increase oxidative stress [26,27], the daily circadian alterations of antioxidant markers under RF exposure have not been demonstrated so far. In this study, we have exposed male rats to 1.8 GHz RF used for mobile phone communications at different times of the day (Greenwich mean time, GMT) to examine the impact on circadian rhythmicity of Mel synthesis as well as GSH-Px and SOD concentrations. The rationale being that the antenna of mobile phones is held close to the head and hence, may have an effect on the brain and perhaps on the pineal gland, which synthesizes Mel.

## 2. Materials and Methods

### 2.1. Animal Handling

All experiments were performed in accordance with the Institutional Animal Care and Use Committee guidelines of the Soochow University and in agreement with its ethical standards [28]. Forty-two 8-weeks-old adult male Sprague-Dawley rats, each weighing approximately 320 ± 10 gm, were obtained from the laboratory animal center, Soochow University. They were housed in a facility maintained at 25 ± 2 °C temperature, 50% ± 5% relative humidity and 12 h light-dark cycles, 7–19 and 19–7 h (GMT), respectively. The animals were fed a commercial diet (Suzhou Shuangshi Laboratory Animal Feed Science Co., Ltd, Suzhou, Jiangsu, China) and water was provided *ad libitum*.

After four weeks of acclimatization, the animals were divided into seven separate groups, six rats in each group. The animals in the first six groups were exposed to far field, continuous wave 1.8 GHz RF (201.7 μW/cm^2^ power density, near to a total power output of about 200 μW/cm^2^ from today’s mobile telephones, which was five times of controlling limits (40 μW/cm^2^) for electromagnetic environment in China in GB 8702–2014) at six specific times during the 24 h light-dark cycle (3, 7, 11, 15, 19 and 23 h GMT, Greenwich mean time), respectively, for 2 h per day for a total of 32 days [12,29]. The animals in the seventh group were kept in a similar RF exposed facility but without RF transmission, for 24 h a day for a total of 32 days. All animal handlings during the dark cycle were carried out using a dim red light (0.1 lux).

### 2.2. RF Exposure Facility and Dosimetry

**Figure 1:**
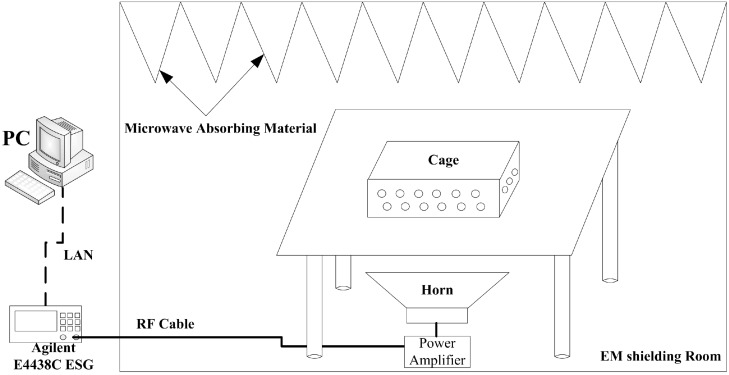
Schematic diagram of 1.8 GHz microwave exposure system.

An in-house built facility was used for 1.8 GHz RF exposure. It consisted of a signal generator (E4438C ESG, Agilent Technologies Inc., Santa Clara, CA, USA) generating RF signals, a power amplifier (SN1020, HD Communications Corp., Ronkonkoma, NY, USA) amplifying RF signals, and a horn antenna positioned to emit RF vertically. A computer was used to control the E4438C by LXI (LAN eXtension for Instrumentation) and log in the exposure information automatically and continuously. In order to avoid the RF reflection, the microwave absorbing materials were hitched on the top side of the facility (Figure 1).

During RF exposure, rats were group housed six animals per cage (466 mm × 315 mm × 210 mm), which was positioned on a table, at a position that provided the required power density of 201.7 μW/cm^2^. The sham controls were treated in an identical way as the exposed groups, except for RF transmission. The power density was measured with a field strength meter (PMM8053A, PMM, Milano, Italy), and was homogeneous when the animals were moving freely during exposure.

**Figure 2:**
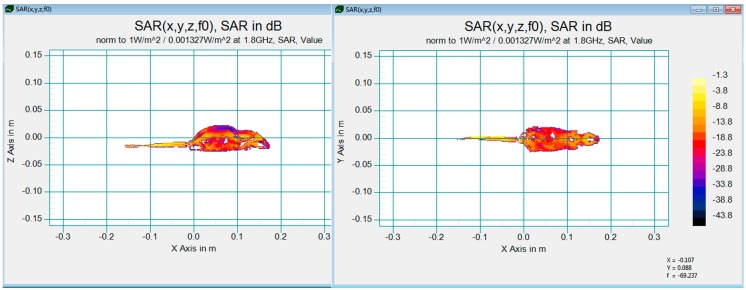
SAR distribution in a phantom 198 g male rat, front and top slice.

The special absorption rate (SAR) was evaluated and computed with a 3D 198 gm male rat model (IT’IS Foundation, Zürich, Switzerland) using a 3D-FDTD full-wave electro-magnetic simulation software, SPEAG SEMCADX_14.8 (SPEAG, Zürich, Switzerland). The FDTD analysis indicated that the numerical whole body SAR distribution was 0.0145W/kg with reference input power density 0.001327 W/m^2^ as shown in Figure 2. Due to the differences of input power and the rat model, the actual whole body SAR was calculated with a correction calculation expressed in Equation (1) shown below [30].

(1) $$\frac{SAR_{E}}{SAR_{s}}=\frac{P_{E}}{P_{s}}\times \frac{W_{s}}{W_{E}}$$

While SAR_S_ is the mean whole body SAR calculated by simulation in SEMCAD, P_S_ and W_S_ are the input power density and weight of a 198 g rat model, SAR_E_ are the mean whole body SAR of rats in the experiments, and P_E_ and W_E_ are the input power density and mean weight of rats in RF groups. As mentioned above, SAR_E_ are approximately 0.05653 W/Kg calculated in Equation (1). SAR_E_ is lower than the threshold of whole-body SAR recommended by Non-Ironizing Radiation Protection guidelines [8], so the non-thermal effect inside the rats in RF groups played an important role in the experiments.

### 2.3. Measurements of Antioxidants

On the 32nd day, immediately after the last 2 h RF exposure, from each rat, 0.1 ml blood was sampled at 5, 9, 13, 17, 21 and 1 h in the next day (GMT) and these were designated as RF3, RF7, RF11, RF15, RF19 and RF23, respectively (indicating the actual time of RF exposure at 3, 7, 11, 15, 19 and 23 h GMT). From each sham-exposed rat, blood samples were also collected at the same time as those in RF-exposed rats mentioned above. Plasma was separated from each blood sample to determine the concentrations of antioxidants, Mel, GSH-PX and SOD.

**Melatonin**: Mel concentrations were evaluated using a commercially available radioimmunoassay (RIA) kit (Immuno-Biological Laboratories, IBL, Hamburg, Germany) according to the manufacturer’s procedure as described earlier [31].

**Glutathione Peroxidase (GSH-Px) and Superoxide Dismutase (SOD)**: Commercially available ELISA kits (Shanghai Yili Biotechnology Co. Ltd., Shanghai, China) were used to determine the levels of GSH-Px and SOD in aliquots of the same plasma samples from each rat by combining horseradish peroxidase (HRP) labeled antibody and TMB (3,3’,5,5’tetramethylbenzidine), and Multiskan GO Microplate Reader (Thermo Fisher, Waltham , MA, USA) at 460 nm [32].

### 2.4. Statistical Analysis

The concentrations of Mel, GSH-Px and SOD obtained at the specified times were fitted by the least squares method for population-mean cosinor analysis and expressed as in Equation (2).

(2) F(t)=M+Acos(ωt+φ)

where F stands for a fitted cosine function, M is the median of the rhythm, A represents the amplitude of the rhythm, ω is the radial frequency (15°/h) of the circadian rhythm, and φ is the peak phase of the rhythm called acrophase. The F-test was used in the Zero-amplitude test to analyze the significance of the circadian rhythm [33]. Cosinor analysis was also performed to fit data using the circadian rhythm fitting software (Departments of Toxicology, School of Public Health, Soochow University, Suzhou, Jiangsu, China). All mean ± standard error data for Mel, GSH-Px and SOD in sham-exposed control animals were compared with those in RF-exposed rats using one-way analysis of variance (ANOVA) and *F*-test and a *p* value of <0.05 was considered significant difference between groups.

## 3. Results

### 3.1. Circadian Rhythm in Sham Exposed Rats

**Figure 3:**
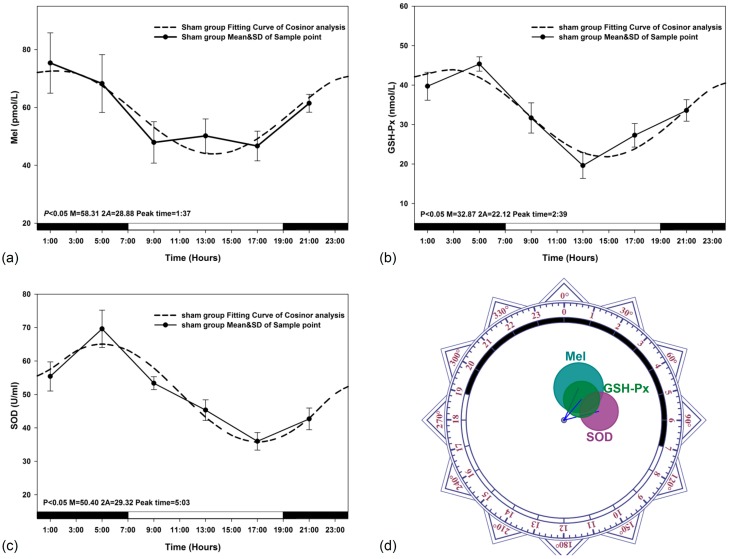
Sham-exposed rats. The best-fitting curves (means ± standard error) determined for Mel (**a**); GSH-Px (**b**) and SOD (**c**); Y-axis represents Mel/GSH-Px/SOD concentration in the plasma; x-axis represents the Time during the 24 h light-dark cycle; (**d**) Zero-amplitude test for circadian rhythms of Mel, GHS-Px and SOD concentration. The circles represent the 95% joint confidence intervals of amplitude and acrophase. The pole (zero-point) of the polar coordinate was not overlapped by the circles, namely the zero amplitude hypothesis was rejected because of *p* < 0.05, which indicates a distinct circadian rhythmicity of Mel, GSH-Px and SOD.

The data obtained for circadian rhythmicity, from zero-amplitude test, for Mel, GSH-Px and SOD levels in sham-exposed animals were presented in Figure 3. The peak levels from fitting curves in Figure 3a–c were observed to reach at 1:37, 2:39 and 5:03 (GMT), respectively. The results of Zero-amplitude tests in Figure 3d have also indicated that the circles of confidence interval were not over-lapping with the pole (Zero-point) to reject the zero amplitude hypothesis, which indicated separate and distinct circadian rhythms for Mel, GSH-Px and SOD levels in the plasma.

### 3.2. Alterations in the Circadian Rhythms of Anti-Oxidant Markers in RF-Exposed Rats

**Figure 4:**
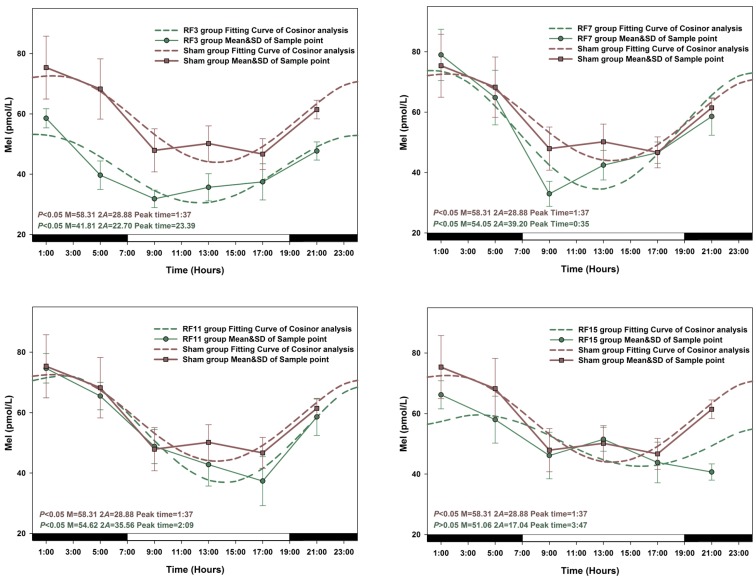

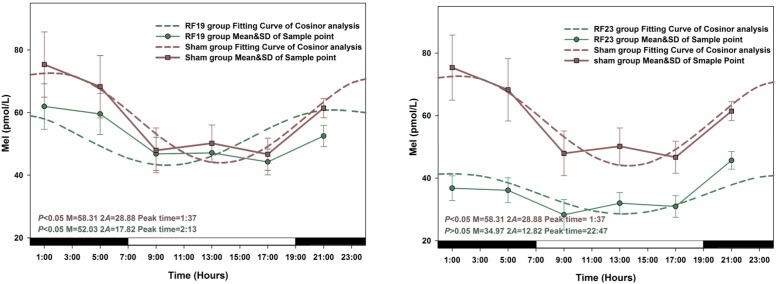
The best-fitting curves (means ± standard error) determined for Mel in RF and Sham-exposed rats. Y-axis represents Mel concentration in the plasma; x-axis represents the Time during the 24 h light-dark cycle. For time-effect, p values from Zero-amplitude test, as well as the median (M), the double amplitude (2A) and the time of peak phase (Peak time) of the 24 h cosine curves, are listed. The circadian rhythm is regarded as significant when *p* < 0.05.

**Figure 5:**
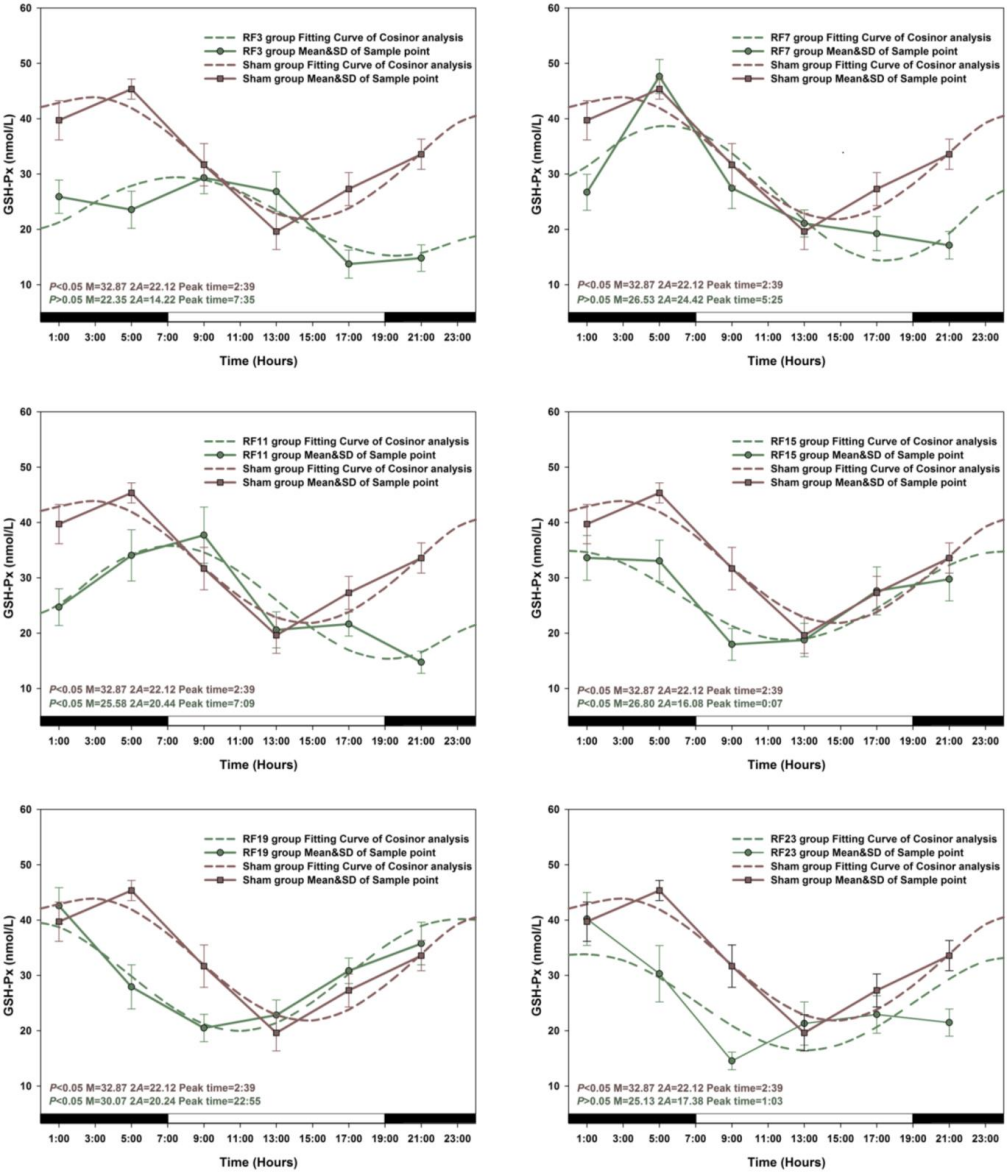
The best-fitting curves (means ± standard error) determined for GSH-Px in RF and Sham-exposed rats. Y-axis represents GSH-Px concentration in the plasma; x-axis represents the Time during the 24 h light-dark cycle. For time-effect, *p* values from Zero-amplitude test, as well as the median (M), the double amplitude (2A) and the time of peak phase (Peak time) of the 24h cosine curves, are listed. The circadian rhythm is regarded as significant when *p* < 0.05.

**Figure 6:**
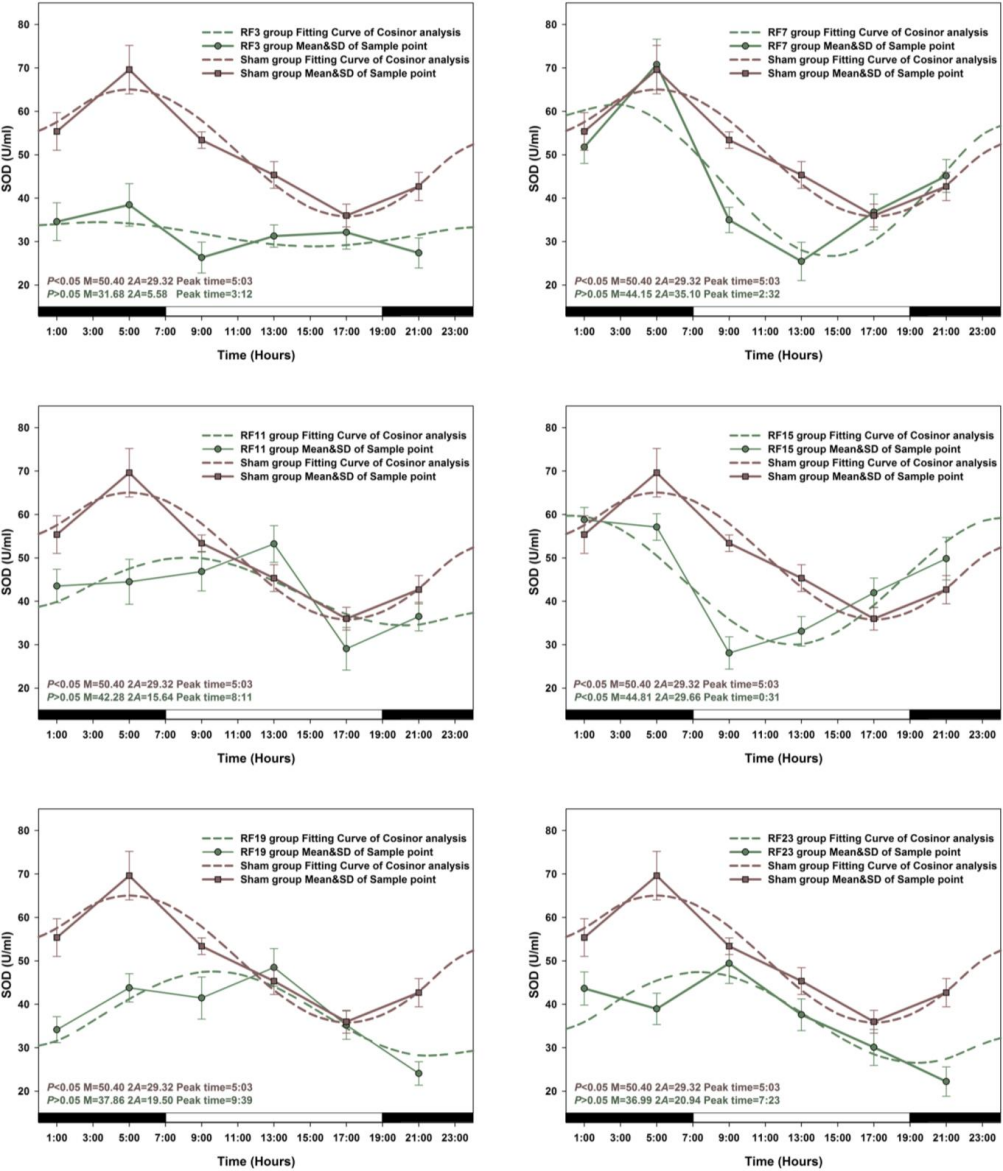
The best-fitting curves (means ± standard error) determined for SOD in RF and Sham-exposed rats. Y-axis represents SOD concentration in the plasma; x-axis represents the Time during the 24 h light-dark cycle. For time-effect, p values from Zero-amplitude test, as well as the median (M), the double amplitude (2A) and the time of peak phase (Peak time) of the 24h cosine curves, are listed. The circadian rhythm is regarded as significant when *p* < 0.05.

The peak time and amplitude changes of Mel, GSH-Px and SOD levels in all groups of rats exposed to RF were significantly different compared with sham-exposed group animals. When a Zero-amplitude test was conducted, the circadian rhythm of Mel secretion was significantly changed in RF15 and RF23 exposed rats (*p* > 0.05), the sharpest variation on amplitude and peak time was observed in the RF23 group. Similarly, the most pronounced amplitude change for GSH-Px and SOD levels was observed in RF3 rats respectively (Figure 4, Figure 5 and Figure 6).

### 3.3. Alterations in Level of Anti-Oxidant Markers Markers in RF Groups

The concentrations of Mel, GSH-Px and SOD in sham-exposed animals and in six RF exposed (RF3, RF7, RF11, RF15, RF19 and RF23) group rats are presented in detail in Figure 4, Figure 5 and Figure 6. Compared with the concentration of Mel, GSH-Px and SOD in sham-exposed animals, the levels in every RF-exposed group rat was not consistent. Most significant decreases in Mel concentrations were in RF3 (*p* < 0.05 at 1:00, 5:00, 9:00, 13:00, 21:00, respectively) and RF23 (*p* < 0.05 at 1:00, 5:00, 9:00, 13:00, 17:00, 21:00, respectively) groups. For the two indicators GSH-Px and SOD, the most profound decreases resulting from RF exposure appeared in RF3 groups compared with sham group (GSH-Px, *p* < 0.05 at 1:00, 5:00, 13:00, 17:00, 21:00, respectively; SOD, *p* < 0.05 at 1:00, 5:00, 9:00, 13:00, 21:00, respectively).

### 3.4. Sensitive to RF for Plasma Anti-Oxidant Functional Markers in Circadian Rhythm

**Figure 7:**
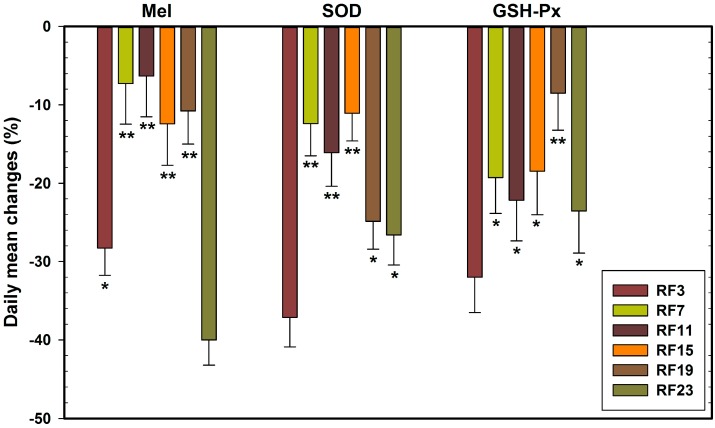
Percentage changes in the daily mean of Mel, GSH-Px and SOD in the plasma of sham-exposed rats and those exposed to 1.8 GHz RF at 3, 7, 11, 15, 19, and 23 h GMT for 2 h/per day for 32 consecutive days. Note: (%) = ((Mean_RF_ − Mean_Sham_)/Mean_Sham_) × 100%.

The % changes in concentrations of Mel, GSH-Px and SOD in RF-exposed rats, during the 24 h period, were calculated using the formula ((group mean in RF-exposed rats–group mean in sham-exposed rats)/group mean in sham-exposed rats) × 100. The calculated values are presented in Figure 7. The data again showed consistency: (a) the % levels of Mel were decreased in RF23 rats more obviously than other RF rats (−40.02); (b) % level of GSH-Px was decreased mostly in RF3 rats (−37.14); (c) % level of SOD was decreased steeply in RF3 rats (−32.01). Thus, the three anti-oxidant markers (Mel and two antioxidases) were sensitive to RF exposure at 23:00, 3:00 (GMT), respectively.

## 4. Discussion

There were several controversial reports in the peer-reviewed scientific literature between exposure to RF frequencies used for mobile communications and adverse human health effects: these include increased genotoxicity, brain tumors and neurodegenerative diseases [34,35,36]. While the cellular mechanisms underlying such effects have not been completely elucidated, one of the putative mechanisms proposed for the observed effects was the induction of ROS/free radicals in RF-exposed cells [26,27]. There is well-documented evidence indicating that ROS induces oxidative damage in major cell macromolecules such as lipids and nucleic acids, which have been implicated in tissue injury [37]. However, cells have developed inherent protective measures by synthesizing antioxidant enzymes such as GSH-Px, SOD, CAT, *etc.* [38]. Thus, the biological antioxidant capacity was suggested to be dependent upon such antioxidant enzymes. Significant decreases in the activity levels of these antioxidant enzymes have been reported in rats exposed to 1.8 GHz RF resulting in increased oxidative stress. There is also ample evidence suggesting that Mel synthesized in the pineal gland in the brain performs the function of antioxidant [39,40]. In experimental animals, pinealectomy was shown to disrupt the circadian rhythm of Mel synthesis leading to decreased antioxidant levels [41] and thus, antioxidant capacity [42]. In a previous study, we reported a robust circadian rhythm for melatonin in rat blood plasma with peak synthesis observed at ZT19 (GMT 2) [43].

Conflicting observations have been reported in the scientific literature on the effect of RF exposure on Mel synthesis. Mel levels were reported to be decreased after RF exposure in Djungarian hamsters and humans at 22:00–0:00 GMT [21,44,45]: these observations were not confirmed in subsequent studies [46,47]. The contradictory results may be due to the differences in electromagnetic fields frequency and power density used in different experiments. The results obtained in the present study indicated significantly decreased levels of Mel in rats exposed to 1.8 GHz RF compared to those in sham-exposed rats. Furthermore, among the six different groups of rats, the levels of Mel were significantly decreased when RF exposures were given at GMT 23:00 and 3:00 when the levels should have been at the highest: thus, the data indicated alteration in amplitude and peak phase of Mel synthesis. Several investigators have reported decreased levels of antioxidant enzymes including GSH-Px and SOD in animals exposed to RF [48,49,50] while others claimed an increase in such levels [51]. The results of the present study showed decreased levels of GSH-Px and SOD levels in rats exposed to 1.8 GHz, especially at 3:00 GMT (RF 3). In addition, the circadian rhythm of GSH-Px and SOD were disrupted in RF-exposed rats, with a distinct disorder of peak phase (from GMT 2:39 to 7:35 or from GMT 5:03 to 3:12 respectively).

Since the three antioxidant functional markers, Mel, GSH-Px and SOD were sensitive to RF exposure at 23 and 3 h (GMT), to explore the more sensitive indicators of RF exposure on antioxidant functional, percentage changes in the daily mean in six time-piont RF exposed group are shown in Figure 7. It can be seen from Figure 7 that the rats in the RF23 group presented the maximum change in Mel concentration (−40.02%) compared to the sham-exposed group. In addition, rats in the RF23 group showed a higher rate of change in Mel levels (−40.02%) compared to those in GSH-Px and SOD levels, −37.14% or −32.01%, respectively, in the RF3 group. The antioxidant enzyme activity rhythm is dependent on the Mel circadian rhythm [52], and it can be speculated that Mel is more sensitive to RF exposure than the other two anti-oxidant markers, SOD and GSH-Px at respective sensitive time. In a previous study, the significant decrease in melatonin levels also occurred near its peak time when rats were exposed to light pulse [53]. The results of these studies suggest that the same regulatory mechanisms may be involved in the alteration of Mel rhythms induced by environmental change of RF or light pulse.

Although the adverse effects on the circadian rhythmicity in Mel and antioxidant enzymes (SOD and GSH-Px) were observed after 1.8 GHz RF exposure in this experiment, it is of interest for us to examine the signaling mechanism of RF exposure on the circadian rhythmicity of anti-oxidant markers. In our experiment, Mel is more sensitive to RF exposure than the other two anti-oxidant markers, SOD and GSH-Px. Mel is synthesized by the pineal gland in the brain in a circadian rhythm which is synchronous with clock gene expression in the pineal gland, as previously reported by our group [54]. The endogenous clock oscillations rely upon genetic mechanisms involving clock genes coding for transcription factors working in negative and positive feedback loops [55]. A major positive loop consists of the basic helix-loop-helix/PAS-type transcriptional activators BMAL1 and CLOCK or its paralog NPAS2, while two cryptochrome (CRY) and three period proteins (PER) are involved in the negative control of the oscillator [56]. Recently, a CRY-dependent pathway was reported to be involved in disrupting negative geotaxis in Drosophila by electromagnetic field [57]. Mel synthesis is also controlled by clock genes [55], though whether CLOCK genes play any role in the adverse effects of RF has not, to our knowledge, been studied yet.

## 5. Conclusions

This study provides evidence implicating circadian rhythmicity in Mel and the antioxidant enzymes SOD and GSH-Px, as well as the effects of RF exposure on the alterations in antioxidant markers. The adverse effects of RF radiation are present in both the average daily levels of antioxidant markers, and their alterations in a 24-h period (circadian rhythm). Although this study clearly shows that RF exposure induces antioxidant circadian rhythm changes in rats, the next work will be mostly performed to study molecular mechanisms of the regulation of circadian rhythm by clock-controlled genes.
